# Advances in Cystic Fibrosis Research in Qatar: A Commentary

**DOI:** 10.3390/jpm13030448

**Published:** 2023-02-28

**Authors:** Samer Hammoudeh, Ibrahim A. Janahi

**Affiliations:** 1 Research Affairs, Academic Health System, Hamad Medical Corporation, Doha P.O. Box 3050, Qatar; 2 Medical Education, Sidra Medicine, Doha P.O. Box 26999, Qatar; 3 Pediatric Pulmonology, Pediatric Medicine, Sidra Medicine, Doha P.O. Box 26999, Qatar

**Keywords:** cystic fibrosis, Qatar, CFTR, I1234V

## Abstract

Cystic fibrosis is a genetic disorder caused by a Cystic Fibrosis Transmembrane Conductance Regulator (CFTR) gene defect. Many across the globe suffer the debilitating symptoms. The aim of this commentary is to briefly cover various aspects related to the disease in the Arab world and then in Qatar.

## 1. Introduction

Cystic fibrosis is a rare, life-restricting, genetic disorder, with multi-organ involvement [1]. Due to a defect in the Cystic Fibrosis Transmembrane Conductance Regulator (CFTR) gene, approximately 70,000–100,000 patients worldwide currently suffer a wide range of symptoms [1,2]. The classical type is manifested by chronic pulmonary inflammation and infection, male infertility, pancreatic exocrine insufficiency, and other comorbidities [2]. The clinical spectrum of some other variants remains unknown due to the lack of studies on these rare cases [1]. Over 2000 mutations currently exist [1,2,3]. The most common CFTR mutation is F508del, which is involved in 80% of cases worldwide [4]. As a result of advances in the diagnosis and management of such patients, the life expectancy of these patients has improved. Reports show a median survival age of up to 48 years in the USA, and 53 years in Europe and Australia [5]. Variations in the clinical course across the globe have been observed. This is due to a number of factors, including access to treatment, late diagnosis, environmental factors, socioeconomic status, and genomic background [2]. The CFTR2 database provides information on CF patients globally [6].

## 2. Arab World

The median survival age of patients in the Arab world ranges from 10 to 20 years [7]. Incidence and prevalence estimates in the Middle East region report a rate of 1/2000–5800 and 1/30,000–50,000, respectively [7]. Due to a high rate of consanguinity, it is suspected that these numbers come short of reality, with many cases being undiagnosed [8]. The clinical outcome for these patients is poor. This is due to various factors, including the lack of specialized centers and late diagnosis [8].

Research on cystic fibrosis across the Arab region has been scarce. A recent systematic review has identified only nine countries that have reported any incidence/prevalence figures in relation to cystic fibrosis patients. The authors of the review reported an apparent lack of longitudinal, quality of life, and life expectation studies [9]. Another systematic review covering the spectrum of mutations among Arabs reported that the most prevalent mutation among Arabs is the c.1521_1523delCTT (p.Phe508del), which was reported in 14 countries. A total of 18 mutations were unique to Arab patients, while 12 were common mutations, and 85 were shared mutations [10]. 

The authors of the latter review recommended further epidemiological studies, especially in regions where consanguineous marriages are common, along with those countries that do not currently have any reports on the CFTR gene mutations. The authors also highlighted the crucial need for standardized diagnostic methodologies for cystic fibrosis across the Arab region, as this would lead to improvement in the detection of CFTR mutations [10]. Other recommendations for the region include the diligent reporting of new cases along with their clinical outcomes, and establishing neonatal screening programs [8].

## 3. Qatar

In Qatar, a review published in 2019 reported the presence of 82 cases (34 adults and 48 children) [11]. The CFTR I1234V mutation is predominantly involved in pediatric cases [12], and was first introduced in 2001 in families with high consanguinity [13]. More recently in 2021, the second gene mutation was identified in Qatar when two children were detected with the homozygous CFTR 1521_1523delCTT (p. Phe508del) mutation [14]. The presence of other mutations was also reported, including the homozygous N1303K [15] and the 1525-1G  >  A [16]. The majority of cases have mild to moderate symptoms [17], are pancreatic sufficient [18], and have low fractional exhaled nitric oxide levels [19]. *Pseudomonas aeruginosa*, *Haemophilus influenza*, and *Staphylococcus aureus* are common pathogens among these patients [20]. A recent study showed that patients colonized with *Pseudomonas aeruginosa* have higher sputum and plasma levels of neutrophil elastase (which play a role in airway inflammation) when compared to those patients without *Pseudomonas aeruginosa* [21].

In relation to screening, the launch of the national newborn screening program in 2003, along with a national premarital genetic screening program in 2009, has been beneficial for the population in Qatar in relation to screening for genetic disorders, including cystic fibrosis. The national newborn screening program is based on a partnership with the University Children’s Hospital of Heidelberg, Germany. It aims to detect metabolic and endocrine disorders, and it has shown a higher incidence of inborn errors of metabolism in Qatar when compared to Germany. The premarital screening program screens for hemoglobinopathies, classical homocystinuria, cystic fibrosis, and spinal muscular atrophy [22]. 

## 4. Future Directions

A survey that sampled 524 cystic fibrosis researchers worldwide showed that CFTR modulators, CFTR modulator combinations, and fixing/replacing the CFTR gene are all viable options in treating cystic fibrosis in the future [23]. Another survey highlighted five research priority areas. These are transplantation, CFTR modulators, *Pseudomonas aeruginosa*, *Burkholderia cepacian*, and *allergic bronchopulmonary aspergillosis* [24]. A third survey identified top 10 research priorities based on a James Lind Alliance methodology [25].

Developing a registry for CF patients in Qatar remains a dire need in order to facilitate future efforts related to the various aspects of disease management and control [11]. This can also be expanded on at the regional level by establishing a registry across Arab countries, which, in turn, would reflect on patient care and overall prognosis [10]. In the future, CRISPR remains a promising technique for replacing or altering the faulty CFTR gene [23,26], along with research involving organoids [27,28] and dietary flavonoids [29,30]. Finally, longitudinal, interventional, and quality of life studies on Qatari patients represent an area for possible research in the future, and, indeed, there is an urgent need for further research that involves patients in Qatar. 

## Data Availability

Not applicable.
